# A review of crime prevention activities in a Japanese local government area since 2008: Beautiful Windows Movement in Adachi Ward

**DOI:** 10.1057/s41300-021-00118-w

**Published:** 2021-06-01

**Authors:** Kimihiro Hino, Themis Chronopoulos

**Affiliations:** 1 Department of Urban Engineering, Graduate School of Engineering, The University of Tokyo, Tokyo, Japan; 2 Department of Political and Cultural Studies, Swansea University, Swansea, UK

**Keywords:** Crime prevention through environmental design, Broken windows theory, Image, Citizen participation, CCTV, Social capital, Tokyo

## Abstract

There have been limited reports on crime prevention policies by local governments in Japan, which is one of the safest countries in the world. This article reviews crime prevention policies in Adachi Ward, which used to have the highest crime rates in Tokyo. The government of Adachi Ward introduced the “Beautiful Windows Movement” (BWM) in 2008. Based on BWM, Adachi Ward implemented various programs and interventions based on two different aspects: the aspect of preventing minor crimes and disorder in partnership with the police referring to the Broken Windows Theory and the aspect of literally making the Ward beautiful in cooperation with citizen volunteers. After 11 years of implementing BWM, the number of recorded crimes in Adachi Ward declined the most in Tokyo and residents’ sense of security improved significantly. This case study highlights the advantage of the duality of BWM to both reduce crimes and improve residents’ sense of security.

**Supplementary Information:**

The online version contains supplementary material available at 10.1057/s41300-021-00118-w.

## Introduction

### Local government and crime prevention

Local governments take responsibility for planning and delivery of crime prevention due to their ability to mobilize local resources; to build partnerships, and to understand local problems (Clancey et al. 2012). Not only do local governments implement crime prevention measures in an effort to serve their constituents, but they are also responsible for the image that their municipality exhibits. High crime rates, especially in urban areas, have the possibility of affecting that image negatively; therefore, it is imperative for local governments to take crime prevention measures that make residents and visitors feel safer. However, relatively little scholarship has focused on localized form of crime prevention (Clancey and Metcalfe 2020). Moreover, to date, there has been a limited focus on crime prevention measures at the local government level in Japan. Although a few studies have reported specific measures for crime prevention in Japan (Komiya 2011; Hino and Schneider 2013; Hino 2018), none has comprehensively reported crime prevention policies by Japanese local governments.

In the first decade of the twenty-first century, the Adachi Ward, one of Tokyo’s 23 Special Wards, experienced the highest crime rates in central Tokyo and was perceived as a crime-ridden area. In 2008, the government of Adachi Ward adopted policies under the heading “Beautiful Windows Movement” (BWM). BWM was influenced by the Broken Windows Theory (Wilson and Kelling 1982) and sought to improve the image of Adachi Ward by tackling crime and improving high profile public spaces.

### Crime situation and countermeasures in Japan

Since the end of World War II (when the authorities began to record crime) to the 1990s, the number of crimes in Japan ranged between 1.3 and 1.5 million per year. During the same period, the population of Japan increased steadily from 72 to 123 million. Crime began to surge in the late 1990s and reached a peak of 2.85 million per year in 2002. Similarly, the number of crimes recorded in Tokyo had been stable in the range of 200,000 to 250,000 per year from the 1960s to the 1980s; it increased sharply in the late 1990s and exceeded 300,000 in 2002.

In 2003, the central government established the Ministerial Conference on Measures Against Crime led by the prime minister and included all the cabinet members (Hino et al. 2018). The Ministerial Conference came up with the “Action Plan for Realizing a Powerful Society Against Crime.” In the action plan, broken windows policing was explained:

In the USA, the “Broken Windows Theory” was proposed as an explanation of the progress of disorder in the community. If a broken window is left unattended, another window will then be torn, eventually ruining the entire building, the entire community. In this way, if a small violation is left untreated, a sense of disorder is gradually created, which leads to a large deterioration of public security. In New York City, USA, in order to restore public security, police cracked down on small violations and took steps to erase subway graffiti. While these are just a part of the improvement of a social environment where crime is unlikely to occur, the crime situation in the city has dramatically improved under these efforts (Ministerial Conference on Measures Against Crime 2003).

This explanation was based on Wilson and Kelling's argument that neighborhoods with greater physical disorder give the impression that community controls have broken down and were more vulnerable to criminals (Wilson and Kelling 1982). It also introduced the public policy aspects of broken windows policing implemented citywide in New York during the Rudolph Giuliani administration (1994–2001) (Chronopoulos 2011, 2020).

In the same year, the National Police Agency (NPA) and related ministries in Japan issued a document urging municipalities to develop crime-resistant communities based on crime prevention through environmental design (CPTED) (Cozens et al. 2005). The document emphasized the importance of properly managing and maintaining the physical environment such as houses, schools, roads and parks in order to deter crimes in the neighborhood. Thus, from 2003 onward, more municipalities in Japan have used features of the broken windows theory and the idea of CPTED to address crime and Adachi Ward in Tokyo was one of them, as described later. It was around that time (in 2002) that the Metropolitan Police Department (MPD) installed as many as 50 CCTVs in Kabukicho, Shinjuku, one of the largest nightlife district sin Tokyo as a precedent for other city centers throughout Japan (Murakami Wood et al. 2007).

Since the mid-1990s, NPA has advocated greater local community involvement to prevent crime and has pursued the building of social capital as a crime prevention strategy, recognizing that the police could not solve the crime problem alone (Leonardsen 2006). Traditionally, neighborhood associations (NHAs), which collectively conduct a range of activities such as cleaning the neighborhood and holding events and festivals based on social capital, play a role in crime prevention and promote a sense of security and familiarity (van Houwelingen 2012; Herber 2018). In this context, following increasing opportunities for voluntary crime prevention activities, in December 2004 the police started to issue a license to volunteers who patrolled areas with a car equipped with a blue-light (police cars are equipped with a red light in Japan). In general, the rise in crime rates in the late 1990s and early 2000s urged the police to revise its business goals; one of these revisions included an improved sense of security. A document issued by the National Public Safety Commission and the NPA stated the following:

As a result of efforts to restore public security in cooperation with related organizations, the number of recorded crimes has decreased for two consecutive years since 2003, …but compared to this, the sense of security of the public has not improved (National Public Safety Commission 2005).

After 2002, the number of recorded crimes in Japan continuously declined. In 2019, it reached the figure of 749 thousand nationally and 105 thousand in Tokyo. This is the lowest crime rate since the beginning of crime statistics (National Police Agency 2020). Currently, Japan is one of the safest countries in the world (OECD 2020). However, little has been reported on what crime prevention policies have been implemented by local governments in Japan.

## Case study site: Adachi Ward, Tokyo

### Overview of Adachi Ward

Adachi Ward is a municipality located in the northeastern part of central Tokyo and is surrounded by rivers on all sides, covering an area of 53.25 km2. Adachi Ward is one of the 23 Special Wards of central Tokyo. Central Tokyo where the wards are located is part of Metropolitan Tokyo, which is one of the 47 prefectures in Japan (something similar to a state in the USA or a province in Canada). Prefectural police departments are set up in each prefecture under the supervision of the NPA, and the MPD has jurisdiction over Tokyo. Adachi Ward has a population of 693,411 (fifth among the 23 Wards) and comprises of 356,641 households (as of May 1, 2020). The population of Adachi Ward has been increasing due to strong development pressures, especially after the opening of two new train lines, the Tsukuba Express and the Nippori-Toneri Liner in 2005 and 2008, respectively.

The Ward had the greatest number of recorded crimes in central Tokyo for four consecutive years from 2006 to 2009. Considering the relatively large area and population among the 23 wards, the number of cases in Adachi Ward was not large. Nevertheless, the Ward’s image suffered both locally and regionally. Yayoi Kondo, the female mayor of Adachi Ward since 2007, strongly felt the need to change the “image” of the area. She pointed out that “Adachi Ward is said to be a “dangerous town” [and that] a woman cannot walk even in the daytime. Some people have such an image without having been to Adachi Ward. However, it is also true that Adachi Ward has had the greatest number of crimes among the 23 wards for the fourth consecutive year” (Kondo 2010). A year later, Kondo said that “most residents don’t think that Adachi Ward is a dangerous city when compared to other cities. However, the image [of a high-crime area] once attached appears to be fixed and cannot be easily dispelled. If that image continues, economic loss will be great. When I became the mayor, I strongly felt that I had to do something about it” (Kondo 2011). As shown in Table 1, Adachi Ward is characterized by a high proportion of the elderly, people in welfare, and public housing. The aging rate has increased by 4.8 percentage points (pp) over the last decade, much higher than the whole 23 wards (3.0 pp). In particular, the percentage in public housing is more than double the average of the 23 wards in central Tokyo. In addition, the financial strength index is relatively low and the number of Ward officials is small. These disadvantages may have contributed to the negative image of Adachi Ward.

**Table 1:** Adachi Ward among Tokyo 23 Wards (population, welfare, government)

	Year	Adachi (Rank in 23 Wards)	23 Wards
Population^a^	2005	624,807 (5)	8,489,653
	2015	670,122 (5)	9,272,740
Aging rate (≥ 65)^a^	2005	19.8 (7)	18.5
	2015	24.6 (2)	21.5
Foreigner rate^a^	2005	2.55 (9)	2.34
	2015	3.22 (12)	3.43
Unemployment rate^a^	2005	3.85 (3)	2.87
	2015	1.91(6)	1.66
Percentage in welfare^b^	2007	3.03 (2)	1.71
	2015	3.74 (2)	2.37
Percentage in public housing (units)^c^	2005	5.27 (1)	2.24
	2019	4.52 (1)	1.97
Voting rate^c^	2005	41.7 (22)	44.1
	2019	55.4 (22)	59.3
Financial strength index^c^	2005	0.33 (22)	0.54
	2019	0.36 (22)	0.54
Government officials/population (‰)^c^	2005	6.27 (23)	9.07
	2019	4.78 (23)	6.09

^a^Population Census (Statistics Bureau of Japan, 2005, 2015)

^b^Welfare and Hygiene Statistics (Tokyo Metropolitan Government, 2007, 2015)

^c^Statistics of Special Wards (2005, 2019)

### Beautiful Windows Movement and Action Plan

Since 2008, the government of Adachi Ward in cooperation with the local community, the police, and other related organizations, has been working to develop the BWM, which aims to create a beautiful, livable city, safe from crime. BWM is influenced by the broken windows theory and its applications in New York City; it is a unique movement that aims to deter crime by giving the impression of “a beautiful town” (Adachi Ward 2016). As the mayor of Adachi Ward argued “I think the Broken Windows Theory as applied in New York City is to change the negative image to zero, but "Beautiful Windows" is not only to make the image neutral but to make it positive” (News Tokyo, 2010). BWM also borrows from the viewpoint of CPTED, which promotes a positive image by routinely maintaining the built environment; this ensures that the physical environment continues to function effectively and transmits positive signals to all users (Cozens et al. 2005). In Japan, the Broken Windows Theory is regarded as a means to enhance the “territoriality” of CPTED: broken windows are a symbol of low territoriality and criminals are believed to visit poorly maintained neighborhoods.

As part of BWM, in April 2010, Adachi Ward and the MPD implemented the “Public Order Restoration Action Plan” (renamed to “Beautiful Windows Movement Action Plan” in 2015), which introduced various crime countermeasures, such as the organizing of crime prevention volunteers and the redesign of roads and parks to conform with basic crime prevention principles (Table A1). The plan stated that CPTED would be applied to roads, parks, and residential developments. Based on the plan incorporating CPTED as well as the Broken Windows Theory, Adachi Ward aimed to create a safer city through the cooperation of crime prevention volunteers formed by local people, schools, and shop owners’ associations, as well as the four police stations in the Ward (Adachi Ward 2016). NHAs were considered to be the main player of BWM. The percentage of households that belong to NHAs in Adachi Ward is 50.9% (Adachi Ward 2019a), a decline from 60.2% in 2007, but still relatively high.

BWM, depicting a virtuous cycle, does not deny the Broken Windows Theory which depicts a vicious cycle. Like the fable of “The North Wind and the Sun,” BWM had the potential of preventing minor crimes and disorder especially in partnership with the police (the north wind), and literally making the Ward beautiful in cooperation with citizen volunteers (the sun). BWM is reasonable in that it tries to improve the image of the ward focusing on crimes and disorders that could be "warning signals" (Innes 2004).

## Research purpose and methods

### Research questions and purpose

As already stated, the model designed to study crime prevention in Adachi Ward is based on (1) crime prevention measures in partnership with the police and (2) crime prevention measures in cooperation with citizen volunteers. The government of Adachi Ward hoped that the combination of the two would reduce the number of recorded crimes and improve the image of the area and the sense of security. We selected as representatives of the category of “crime prevention measures in partnership with the police” the following: measures against bicycle theft and the installation of CCTVs. We focused on measures against bicycle thefts because they represented the highest proportion of crime; the Ward viewed reduction in bicycle theft, which directly links to the reduction in total number of crimes, as a way to dispel the negative image of the Ward (Kondo 2011). As described later, the installation of CCTVs is a measure that Adachi Ward put more effort into than other wards, and the police advised the ward on their location. Then, we selected as representatives of the category of crime prevention measures in cooperation with citizen volunteers the following: crime prevention patrols, streets with flowers project, and certification of “neighborhoods that promote the prevention of crime” (the direct translation from Japanese is “crime prevention promotion neighborhood”). These two categories are our subjects of analysis.

The two key research questions of this study are:

- What interventions and programs exist in Adachi Ward based on BWM?
- To what extent are these programs and interventions responsive to crime problems and citizens’ sense of security?

Through answering these research questions, this article examines the usefulness of BWM derived from the Broken Widows Windows Theory by conducting a case study of Adachi Ward. As already mentioned, to date there has been a limited focus on crime prevention measures at the local government level in Japan. This study will contribute to crime prevention policy research by adding a case study of a Japanese local government, which used to have high crime rates (according to Japanese standards).

### Methods

The research methods adopted for this study include the following: (1) interviews with Ward staff and executives; (2) crime data analysis; and (3) analysis of policy documents. One of the authors has been an advisor of Adachi Ward on crime matters since 2010 and exchanges opinions with the staff of the Risk Management section of the Ward almost monthly. Moreover, the author attends BWM Promotion Strategy Meetings and crime prevention events where he meets the mayor and executives of the Adachi Ward. In addition, in the “neighborhoods that promote the prevention of crime” project, he has joined the Community Development Section of the Ward and participates in workshops with local residents about three times a year.

In the crime data analysis section of this article, we use two sets of data. The first is the recorded number of crimes recorded by the MPD. The second is the citizens’ sense of security since 2007, one year before BWM started. Sense of security has been measured in questionnaire surveys conducted by the Adachi Ward every summer among 3,000 randomly selected citizens aged 20 and over (response rate: 53.0% in 2019). The director of the Risk Management section (temporarily transferred from MPD) has cooperated with the data collection.

## Crime prevention measures in partnership with the police

### Partnership with the Metropolitan Police Department (MPD)

In order to rectify the crime situation and improve the image of the Ward, the local government and the Community Safety Department of the MPD published the “Protocol Promoting the Restoration of Security” on December 21, 2009 (Table A2). This helped to strengthen the relationship between MPD and the Adachi Ward. It was the first time that the MPD, which has jurisdiction over the entire Metropolitan Tokyo, published such a protocol with a specific municipality, suggesting that the MPD was also concerned with the crime situation in Adachi Ward. Centering on the BWM, the Ward has taken measures such as strengthening crime prevention patrols and expanding activities for citizens’ awareness on crime prevention with the cooperation of the MPD and the four police stations in the ward. Based on the protocol, MPD shared crime information with the Ward for the first time as a municipality. For example, the Ward opened a “Crime Map” on the website to inform where crimes had been occurring in order to enhance citizens’ awareness and deter crimes (Adachi Ward 2016). The Ward also sent information on crime occurrence and crime prevention to approximately 52,400 email addresses of pre-registered citizens. Although their personal attributes are unknown, it is thought that most of them are parents of elementary school students and members of NHAs who carry out crime prevention activities.

### Measures against Bicycle Theft

Adachi Ward has been focusing on prevention of bicycle theft because it occupies approximately one-third of recorded crimes in the Ward. The following are the examples of countermeasures against bicycle thefts:

- Operation “Aijo (with affection) Lock”: The staff locks unlocked bicycles without owners’ permission in public parking lots and large commercial facilities a few times a month. The bike owners ask the staff for the lock number, unlock it, and get the lock for free.
- “Gacchiri (firm) Lock” campaign: The staff exchanges weak bike locks with strong ones for free a few times a year on occasions such as festivals where many people gather (Adachi Ward 2016). The campaigns are carried out jointly by the ward and police stations, and the cost of locks are borne by the ward.
- Youth volunteers for bike-theft prevention: High school students and other young volunteers inform their colleagues that they should lock bicycles and that bicycle theft is a crime (because approximately one-third of the victims are teenagers) twice a year in spring and autumn. For example, 56 bike locks were replaced in a public residential estate where bicycle thefts occurred frequently in October 2020.
- Ordinance on safe use of bicycles: An ordinance which prohibits leaving bicycles in designated areas such as around railway stations and requires users to lock or take other appropriate measures (revised in January 2018).

In addition to these, the Ward carries out various programs and interventions collaborating with the police stations and crime prevention volunteers.

### Installation of CCTVs

While the first action plan (2010) included the installation of CCTVs in streets and in public facilities, subsequent plans added installation around elementary schools and in parks. The Tokyo Metropolitan Government actually urged municipalities to install CCTVs in anticipation of the Tokyo 2020 Olympic and Paralympic Games; however, Adachi Ward focused more on CCTVs than other municipalities in Tokyo. The Ward has installed 670 CCTVs in public spaces, especially in local parks, around elementary schools, and busy commercial areas (as of April 2019). Originally, these CCTVs had been set up and managed by each section of the Ward, such as the road section and the park section. As it took up to 10 days to provide information to the police, the system was changed and only one section manages CCTVs. Currently, information is provided on the same day or 2 days after the police makes a request. Responses to breakdowns of CCTVs have also been speeded up, and strategic installation based on the distribution of existing CCTVs has become possible. In addition, 1160 CCTVs have been installed by NHAs in residential and local commercial areas with the subsidy of the Ward. The Ward subsidizes 95% of the installation cost and part of the operation cost; the subsidy amounts to approximately 81 million yen per year.

## Crime prevention measures in cooperation with citizen volunteers

Crime volunteer patrols, “Streets with flowers project,” and certification of neighborhoods are discussed in this section. While aiming at crime prevention, all of them improve the sense of security through developing social capital.

### Crime prevention patrols

Besides crime prevention patrols by private security services entrusted by the Ward, crime prevention volunteers (CPVs) patrol their neighborhood. Residents’ voluntary crime prevention activities in Japan are generally carried out in each neighborhood by members of NHAs, parents and teachers’ associations (PTAs), and other local stakeholders. According to police, 4,500 citizens in 170 groups were registered as CPVs in Adachi Ward (as of the end of 2019).

Activities of CPVs are diverse, including patrol (on foot and using blue-light cars), cleaning neighborhoods, planting flowers, checking risky places, watching out for children walking to/from school, conducting activities to prevent fraud to elderly, and so on. These actions raise residents’ awareness that they have to keep their neighborhoods safe by themselves. The Ward supports them providing tools used in crime prevention activities and volunteer insurance. The Ward also provides financial support for CPVs that conduct patrol more than twice a month to watch for children walking to/from school. The support is up to 100,000 yen per group for buying equipment such as vests, jackets, armbands, clappers, flashlights, and flags (the total is approximately 4 million yen per year). Physical offenses against children are concentrated during this time, and CPVs are expected to prevent such offenses and improve the sense of security (Cabinet Secretariat 2018). In addition, the Ward deploys 11 blue-light patrol cars for the use of citizen volunteers. While the impact of citizens’ activities on crime is difficult to assess, these activities provide opportunities for increased social interaction and social capital (Herber 2014, 2018).

### Streets with Flowers Project

Adachi Ward introduced the “Streets with Flowers Project” in 2013. Residents were given labels to insert in flowerpots when they bought seedlings at cooperating flower shops. Residents then placed the flowerpots with labels in front of their houses and watered them when school children walked to/from school (Hino 2018). From CPTED point of view, the project meant to enhance the surveillance in the area, while at the same time it improved territoriality through the strengthening of the social tie.

The project has contributed to the beautification of the Ward and is considered a symbol of the BWM. Residents who grow the flowers and play a role in crime prevention are called a "Beautiful Partner" of the Ward. Nursery schools, kindergartens, and other children’s facilities participate in the project and fill the city with flowers. Children using these facilities grow sunflowers from seeds and bring their seedling back home to be raised. Staff of these facilities also grow flowers in flower beds and planters in each garden and watch the children while watering or caring it. In 2019, 99 facilities participated in the project. In addition, 16 shop owners’ associations and 213 shops grew tulips and periwinkle plants in front of the stores (Adachi Ward 2020b).

### Certification of “Neighborhoods that promote the prevention of crime”

There are two certification systems in the Ward that are complementary and seek to achieve a safe environment. Originally, Adachi Ward had a certification system, in which a residential estate that had a certain level of crime prevention performance was certified as a “Crime Prevention Design Town.” This was an incentive for developers to follow the “Adachi CPTED Guideline” formulated in April 2011 (Hino and Schneider 2013). However, it was difficult to improve the physical aspects of a neighborhood to meet these criteria unless it was newly developed. Because of this, the Ward added the “neighborhoods that promote the prevention of crime” program, which is a certification system that focuses on the unity of the community members and complements the physical aspects of the neighborhood. The Ward certifies NHAs that formulate a “Charter for safe community building” (see Table 2), which lists crime prevention activities that residents will autonomously carry out, as “neighborhoods that promote the prevention of crime.” The Ward and an NHA aiming to be certified first conduct a “crime prevention audit” consisting of neighborhood walking and map creation and then formulate a charter through an adviser’s lectures and workshops. The Ward supports the activities on the charter of the certified neighborhood by introducing support programs and connecting relevant stakeholders. From 2014–2019, eighteen neighborhoods were certified under this scheme. Five years after formulating a charter, the NHAs can apply for renewal of the certification following self-assessment of achievement levels and, if necessary, revision of the charter.

**Table 2:** An example of a charter shown to participants (Adachi Ward 2019b)

We formulate this charter aiming to create a safe and secure neighborhood for from children to the elderly 1. We patrol our neighborhood using the blue-light patrol car 2. We grow flowers and trees with affection 3. We take a walk watching out for children 4. We lock our bicycle even on our own premises 5. We share information on vacant houses and vacant lots 6. We replace streetlights with LEDs to make our neighborhood brighter	

The expectation is that this kind of community-based collaborative process will help improve participation in crime prevention (Piroozfar et al. 2019). The following are comments of participating residents in the certified neighborhood:

"I am happy to have an opportunity to discuss the neighborhood with other residents,"

"It led to building flower beds in the park,"

"The charter is a checklist for our neighborhood activities."

In that sense, the certification of neighborhoods formalizes local crime prevention activities and provides an additional layer of security.

## Crime trends and citizens’ sense of security

### Crime trends

**Fig. 1:**
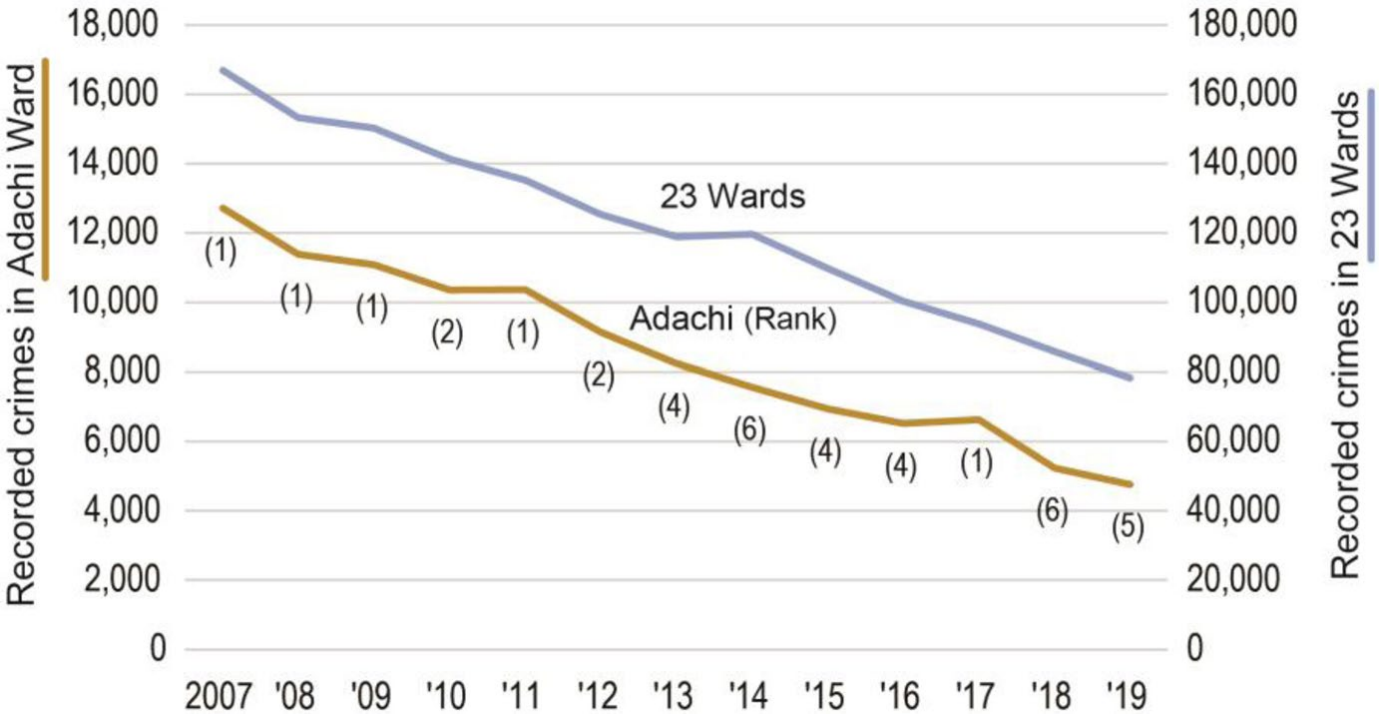
Trend of total recorded crime in Adachi Ward and Tokyo 23 Wards. * The label on the line shows the rank of Adachi Ward in 23 Wards. Source: Metropolitan Police Department (2020)

The number of recorded crimes in Adachi Ward was the highest among the 23 wards in central Tokyo until 2009. In the years that followed, the number declined. In 2010, Adachi Ward no longer had the highest crime rate in central Tokyo and reached the 6th place in 2014 and 2018 (Fig. 1). The number of crimes declined by 62.6 pp from 2007 to 2019 in Adachi Ward. This was the largest decrease in the 23 Wards (crime declined by 53.1 pp in all 23 Wards). By category of crime, burglary declined in particular; the number of bicycle thefts, in which Adachi Ward strengthened its measures, decreased by 54.8 pp from 2007 to 2019 and this was the largest decrease in the 23 wards (Table 3). The significant decrease in felony and other larceny is due to the fact that bag snatching has rarely occurred in recent years (bag snatching is classified as larceny, unless the victim is injured when it is classified as felony). These categories decreased in other wards as well; however, the decrease is noticeable in Adachi Ward where there were more bag snatchings. Overall, crime rates declined substantially in Adachi Ward and the expectation was that the residents’ sense of security would also improve.

**Table 3:** Change in numbers of each crime category in Adachi Ward and the 23 Wards

Year		2008	‘09	‘10	‘11	‘12	‘13	‘14	‘15	‘16	‘17	‘18	‘19
Felony	Adachi	122	67	93	124	120	81	78	83	74	72	48	35
	23 Wards	107	98	81	84	80	75	84	71	65	66	65	63
Violence	Adachi	96	84	77	85	93	80	88	80	81	96	88	76
	23 Wards	95	89	82	81	85	84	86	89	85	82	84	75
Burglary	Adachi	104	73	80	63	56	48	43	36	32	40	28	24
	23 Wards	90	86	71	61	60	60	55	49	40	40	35	32
Bicycle theft	Adachi	93	99	87	90	77	69	69	73	69	67	48	45
	23 Wards	97	101	96	100	89	86	99	88	81	71	61	56
Other larceny	Adachi	83	83	78	81	69	62	52	44	40	39	35	31
	23 Wards	88	86	83	77	70	65	60	56	50	47	44	42
Others	Adachi	91	85	81	75	70	66	60	50	49	51	40	37
	23 Wards	92	86	79	72	70	66	63	58	55	55	51	42
Total	Adachi	90	87	81	81	72	65	59	55	51	52	41	37
	23 Wards	92	90	85	81	75	71	72	66	60	56	51	47

(Number of each category in 2007 = 100)

Source: Metropolitan Police Department (2020)

### Citizens’ sense of security

**Fig. 2:**
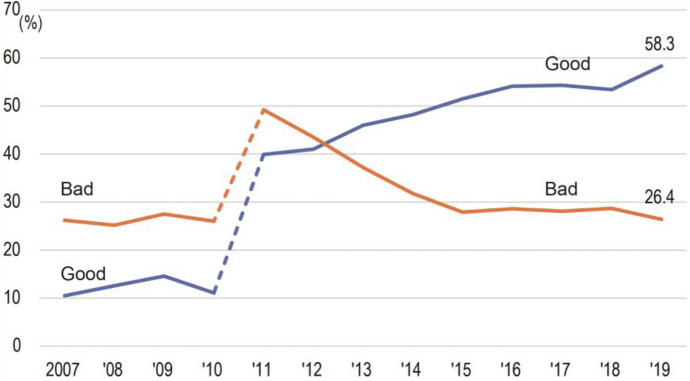
Citizens’ sense of security (Adachi Ward, 2020a). * The question has changed since 2011 (see Table A2)

Sense of security was measured in questionnaire surveys of 3,000 adult citizens randomly selected from the Adachi Ward Basic Resident Register conducted by Adachi Ward every summer. The questions regarding sense of security have changed since 2011 (Table A3). However, despite the changes, in a general sense residents view security as “Good” or “Bad.” As shown in Fig. 2, “Bad” was about 15% higher than “Good” between 2007 and 2010. In 2011 and 2012, the responses got closer to each other and from 2013 onward, the majority of respondents viewed security as “Good.” In the latest 2019 survey, responses of “Good” were more than double those for “Bad”. Since 2014, the reasons why respondents chose “Good” or “Bad” have been asked. The percentage of people who viewed security as improved because of more CCTVs almost doubled; it went from 18.7% to 35.7%. In addition, the percentage of people who chose “Good” because of fewer crimes went from 18.1% to 21.9%, while the percentage of people who chose “Good” because of active voluntary crime prevention patrols decreased from 20.9% to 14.3%. Finally, the percentage of respondents who chose “Bad” because of the number of crimes such as burglary and bicycle theft declined from 60% to 50.4%. Nonetheless, by 2019, 58.3% of respondents viewed the security situation in their neighborhood as “Good” as opposed to 26.4% who viewed it as “Bad.”

## Discussion

This study reported the implementation of various crime prevention measures based on BWM, which resulted in a reduction of crimes and the improvement of citizens’ sense of security in Adachi Ward. Crime prevention measures involved two different types; (1) those in partnership with the police and (2) those to make the Ward beautiful in cooperation with citizen volunteers. The first directly reduced the number of crimes, which improved citizens’ sense of security. It can be said that the strategy of focusing on measures against minor crimes, especially bicycle theft which decreased by 10 pp more than the average of all 23 wards, seems to have worked well. At the same time, it is difficult to quantify and connect various measures with results. This is also apparent from the results of annual surveys, which overall are positive but specifically vary. Still, the large decrease in the number of crimes and the release from the stigma of Adachi being “the worst among the 23 Wards” certainly contributed to the improvement of citizens’ sense of security.

The activities in cooperation with citizen volunteers, which enriched social capital in the neighborhood, contributed to the decrease in the number of crimes, and improved citizens’ sense of security (Matsukawa and Tatsuki 2018). A previous study of Tokyo’s 23 Wards found that social capital has a stronger positive association with residents’ sense of security than actual crime rates in their neighborhood (Hino et al. 2018). Voluntary crime prevention patrols were one of the reasons for a positive sense of security in the result of the Ward’s annual survey; however, the proportion of patrols has decreased in contrast to the increasing proportion of CCTVs. Installation of CCTVs, thus, contributed to the improvement of residents’ sense of security, as well as the reduction in the number of crimes. Takagi et al. (2020) found a negative association between residents’ awareness of CCTVs installed by a municipality and their perceived social cohesion in Ichikawa City, which is adjacent to Tokyo 23 Wards. As they argued, the presence of CCTVs might be accepted as a sign of a lack of social networks to exercise collective crime prevention activities. This could be the case in Adachi Ward, as well. This phenomenon needs to be recognized as a negative aspect of CCTV installation. Since the installation of CCTVs is actually promoted by the cooperation of NHAs and the Ward, it would be necessary to let citizens know the fact and recognize the presence of social capital.

In Japan, crime prevention volunteering is an indispensable part of neighborhood self-governance, and individual participation means the inclusion in a bigger network of people being active in the neighborhood (Herber 2018). However, it is generally true that NHAs and CPVs are becoming less active due to the aging of existing members and the non-enrollment of young households. Moreover, excessive sense of self-governance can lead to the intolerance of others: peer pressure and mutual surveillance of citizens, that functioned during the first wave of the COVID-19 in Japan, instead of mandatory lockdown and police intervention seen in other countries, sometimes caused harassment of potential spreaders. Adachi Ward has adopted “co-creation” as a keyword in its Basic Concept (top-level policy document showing future goals and basic measures of a local government) since 2017 where various local stakeholders solve local issues voluntarily and autonomously recognizing each other's individuality and values. The Ward tends to support them instead of taking the initiative to solve problems. Based on this, not only traditional NHAs but also young people and private companies should be expected to participate in BWM. The “Plus Bouhan” approach that adds aspects of crime prevention to daily activities, in which a diverse range of stakeholders can participate with little burden (Hino 2018), would be useful.

Thus, unlike broken windows policing adopted by the police departments of various cities in the USA in the 1990s (Felker-Kantor 2018; Kohler-Hausmann 2018), Adachi Ward put emphasis on making people feel safer by focusing on the improvement and beautification of public spaces and the involvement of local residents in crime prevention through volunteer patrols, certification systems, and the constant evaluation of conditions. The police does not resort to aggressive policing and does not ticket people for minor violations as a way to reduce serious crime.

### Limitations

Although making an important contribution to existing research by reviewing crime prevention measures at the local government level in Japan, this study has a few limitations. First, we cannot conclude that there was a causal relationship between the crime prevention efforts, the reduction in the number of crimes, and the improvement of citizens’ sense of security. Indeed, this has been the case worldwide, as crime has a structural dimension that depends on local, regional, and national factors. Second, the conclusions of this study cannot be generalized to local governments in other countries because crime prevention policy in Adachi Ward is based on the Japanese criminal justice system and the NHAs rooted in Japanese tradition and culture. In Japanese local governments, the conclusion may be applicable under the leadership of the mayor, taking into account the local context.

## Conclusions

This article reviews crime prevention policies in Adachi Ward, which used to have the highest of crimes in Tokyo. Based on BWM that Adachi Ward introduced in 2008, it implemented various programs and interventions based on two different aspects; the aspect of preventing minor crimes and disorder in partnership with the police referring to the Broken Windows Theory and the aspect of literally making the ward beautiful in cooperation with citizen volunteers. After 11 years of implementing BWM, the number of recorded crimes in Adachi Ward declined the most in Tokyo and residents’ sense of security improved significantly. This case study highlights the advantage of the duality of BWM to both reduce crimes and improve residents’ sense of security.

## Supplementary Information

Below is the link to the electronic supplementary material.

Supplementary file1 (PDF 100 KB)
