# Supplementary material for: A review of crime prevention activities in a Japanese local government area since 2008: Beautiful Windows Movement in Adachi Ward
Source: Crime Prev Community Saf. 2021 Jun 1;23(3):341–57. doi: 10.1057/s41300-021-00118-w (PMC8167388; doi:10.1057/s41300-021-00118-w)
Supplement: Supplementary file 1 — Supplementary file1 (PDF 100 KB) [file 41300_2021_118_MOESM1_ESM.pdf]

Table A1 BWM Action Plan (as of March 2020)

I Promotion of comprehensive crime prevention measures

1 System establishment to promote crime prevention measures

(1) Enforcement of security measures by the united efforts of various stakeholders

(2) Establishment of "Adachi Ward BWM Promotion Strategy Meeting"

2 Accurate survey/analysis of crimes and selection of priority areas

(1) Promotion of accurate crime survey

(2) Prioritized crime categories and geographical areas

(3) Effective crime prevention measures and strengthening crackdowns

(4) Strengthening cooperation between neighboring prefectural police and local governments

(5) Promotion of measures in busy commercial areas

3 Promotion of crime prevention measures by crime category

(1) Measures against burglary

(2) Measures against vehicle (bicycle/motorcycle) theft

(3) Measures against special fraud (transfer/refund fraud, etc.)

(4) Measures against bag snatching

(5) Fostering a social environment that does not allow shoplifting

4 Empowerment of local communities for crime prevention

(1) Audit of neighborhood environment for crime prevention

(2) Planning of a safety and security station

5 Ensuring the safety and security of older adults

6 Strengthening crime prevention activities by volunteers

(1) Support for "Beautiful partners"

(2) Guidance to volunteers

(3) Expansion of volunteers

(4) Implementation of joint patrol

7 Countermeasures against crime by "Beautiful keepers"

(1) Crime prevention measures by "Station keepers"

(2) Enhancement of night time patrols

(3) Raising citizens' awareness for crime prevention using ward-owned vehicles

(4) Measures to prevent smoking in public spaces

(5) Measures to prevent stealing recyclable resources

(6) Measures to prevent illegal dumping

8 Raising awareness and information dissemination

(1) Raising citizens' awareness

(2) Information dissemination

II Promotion of town development that is strong against crime

1 Utilization of CPTED Guideline

(1) Road

(2) Park

(3) Parking lot/bicycle parking

(4) Certification of “Crime prevention promotion neighborhood”

2 Measures against residential burglary

(1) Spread of building parts with high crime prevention performance

(2) Spread of Tokyo crime prevention excellent apartment registration system

(3) Effective use of Building crime prevention advisers of MPD

(4) Improvement of crime prevention performance of houses

(5) Information sharing on special fraud using unoccupied houses

3 Installation of CCTVs

(1) Increasing the number of CCTVs

(2) Installation of CCTVs in public facilities

(3) Installation of CCTVs around public elementary schools

(4) Promotion of installation of CCTVs by local residents and businesses

(5) Installation of CCTVs in parks

(6) Collective management of CCTVs

4 Installation of sensor lights

5 Installation of signboards on the street

6 Environmental improvement of public housing

7 Protection of youth from harmful environment

8 Counterterrorism toward the Tokyo 2020 Olympic and Paralympic Games

III Promotion of the third beautiful town development

1 Building a beautiful city without crime

(1) Fostering social bonds in local community

(2) Beautification of townscape

(3) Promotion of "Streets with flowers project"

(4) Crackdown on disorders that damage the townscape

(5) Various crime prevention and beautification activities

2 Education to improve manners and rules

IV Promotion of Youth Development Project

1 Ensuring the safety of children

(1) Safety education for children

(2) Improvement of environment for the safety and healthy development of children

(3) Installation of CCTVs around public elementary schools

(4) Watching out and patrolling for children

2 Creating a society without delinquency

(1) Promotion of measures to enhance children's normative consciousness

(2) Fostering a social network to watch over children

(3) Recovery support activities to reach out to children

Table A2 Protocol Promoting the Restoration of Security in Adachi Ward

Regarding the security restoration project in Adachi Ward, Adachi Ward and the Metropolitan Police Department (MPD) confirm the following in order to promote the project in cooperation.<br><br>1. Adachi Ward will make all-out efforts to engage in security and restoration projects on both hardware and software sides, including the "Beautiful Windows Movement".<br>2. The Community Safety Department, MPD and the police stations in Adachi Ward will analyze the crime situation, share the information, provide advice on the planning of projects, support crime prevention patrol groups, and provide other necessary cooperation in order to carry out projects smoothly and effectively.<br>3. Adachi Ward and the Community Safety Department, MPD, and the police stations will further exchange information and strengthen mutual cooperation in order to carry out projects smoothly and effectively.<br>4. Adachi Ward and the Community Safety Department, MPD will formulate an action plan to carry out specific security restoration projects.<br>December 21, 2009<br>Yayoi Kondo, Mayor of Adachi Ward<br>Fumio Yamashita, Chief of Community Safety Department, MPD

Table A3 Questions on sense of security in the survey

Year	Questions	Options
-2010	What's a good/bad point to live in your neighborhood? (multiple answers for good/bad, respectively)	Parks and greenery, Relationships in the neighborhood, Convenience for cultural facilities, Parenting and education environment, Convenience for everyday shopping, Prices of commodities, Convenience for exercise facilities, Transportation for commuting to work or school, Convenience for medical facilities, Security, Other, Nothing.
2011-	How do you feel about the security of your neighborhood?<br>Which is the reason you chose good/bad about the security of your neighborhood? (up to two options)	Good, Rather good, Rather bad, Bad, Not sure.<br>**Good:** CCTVs have increased, There is little news about crime, Crimes are decreasing, No one is involved in the crime, Voluntary crime prevention patrol is active, Roads and parks are safely designed, Awareness about crime prevention is improved, Other.<br>**Bad**: There are many violent crimes such as murder, robbery, and arson, There are many crimes such as bicycle theft and burglary, There are many suspicious cases against children, There are few street lights, Young people gathering at late night, There is no sense of solidarity in the neighborhood, Other.
